# PEDL+: protein-centered relation extraction from PubMed at your fingertip

**DOI:** 10.1093/bioinformatics/btad603

**Published:** 2023-11-09

**Authors:** Leon Weber, Fabio Barth, Leonie Lorenz, Fabian Konrath, Kirsten Huska, Jana Wolf, Ulf Leser

**Affiliations:** Center for Information and Language Processing, Ludwig-Maximilians-Universität München, Geschwister-Scholl-Platz 1, München 80539, Germany; Computer Science Department, Humboldt-Universität zu Berlin, Unter den Linden 6, Berlin 10099, Germany; Pathogen Informatics and Modelling, EMBL-EBI, Hinxton, Cambridgeshire CB10 1SD, United Kingdom; Mathematical Modelling of Cellular Processes, Max Delbrück Center for Molecular Medicine, Robert-Rössle-Str. 10, Berlin 13125, Germany; Mathematical Modelling of Cellular Processes, Max Delbrück Center for Molecular Medicine, Robert-Rössle-Str. 10, Berlin 13125, Germany; Mathematical Modelling of Cellular Processes, Max Delbrück Center for Molecular Medicine, Robert-Rössle-Str. 10, Berlin 13125, Germany; Department of Mathematics and Computer Science, Free University Berlin, Berlin, 14195, Germany; Computer Science Department, Humboldt-Universität zu Berlin, Unter den Linden 6, Berlin 10099, Germany

## Abstract

**Summary:**

Relation extraction (RE) from large text collections is an important tool for database curation, pathway reconstruction, or functional omics data analysis. In practice, RE often is part of a complex data analysis pipeline requiring specific adaptations like restricting the types of relations or the set of proteins to be considered. However, current systems are either non-programmable web sites or research code with fixed functionality. We present PEDL+, a user-friendly tool for extracting protein–protein and protein–chemical associations from PubMed articles. PEDL+ combines state-of-the-art NLP technology with adaptable ranking and filtering options and can easily be integrated into analysis pipelines. We evaluated PEDL+ in two pathway curation projects and found that 59% to 80% of its extractions were helpful.

**Availability and implementation:**

PEDL+ is freely available at https://github.com/leonweber/pedl.

## 1 Introduction

Extracting relations between biomedical entities in scientific texts is a versatile technique for enriching experimental results with biomedical context, for instance in pathway curation (Gyori *et al.* 2017). However, current tools actually available for performing relation extraction (RE) at scale are either implemented as research code with fixed functionality (Lee *et al.* 2020), rely on manually defined rules (Gyori *et al.* 2017), or require gold-standard datasets which are costly to produce (Van Landeghem *et al.* 2013). This makes is difficult to apply them in practice, where RE models must be adapted to the specific needs of a project, for instance by restricting the proteins to be considered, the relations to be extracted, or the papers to be analyzed.

We recently introduced PEDL (Weber *et al.* 2020), a versatile RE model combining distant supervision and pre-trained language models (PLMs) that achieves high accuracy without tailored rules or large gold-standard training data (Weber *et al.* 2022). However, the research code published alongside PEDL did neither allow the application of PEDL to user-defined text nor to aggregate, interpret, or filter the extractions. Here, we present PEDL+, a complete re-implementation of PEDL as an easy-to-use, scalable and customizable command-line tool, which allows the application of state-of-the-art RE models to large amounts of text with just a few commands. PEDL+ supports the extraction of seven types of protein–protein associations (PPAs: controls-phosphorylation-of, controls-state-change-of, controls-transport-of, controls-expression-of, in-complex-with, interacts-with, and catalysis-precedes) and 12 types of chemical–protein relations (CPAs: antagonist, agonist, agonist-inhibitor, direct-regulator, activator, inhibitor, indirect-downregulator, indirect-upregulator, part-of, product-of, substrate, and substrate_product-of), is easy to install and use, and can be adapted to only the proteins of interest, only papers on certain topics, and only relations scored above a user-defined confidence threshold. It comes with a detailed tutorial notebook that showcases the different ways in which PEDL+ can be applied. A careful evaluation in two pathway curation projects regarding cell fate decisions in diffuse large B-cell lymphoma showed the overall high usefulness of its results. We compare the functionality of PEDL+ to related tools in Supplementary SM 1 and find that it is the only one based on PLMs, which typically are much more accurate than more traditional machine learning methods (Devlin *et al.* 2019). Additionally, it is much faster than the compared PPA extraction tools.

## 2 Functionality

**Figure 1.**
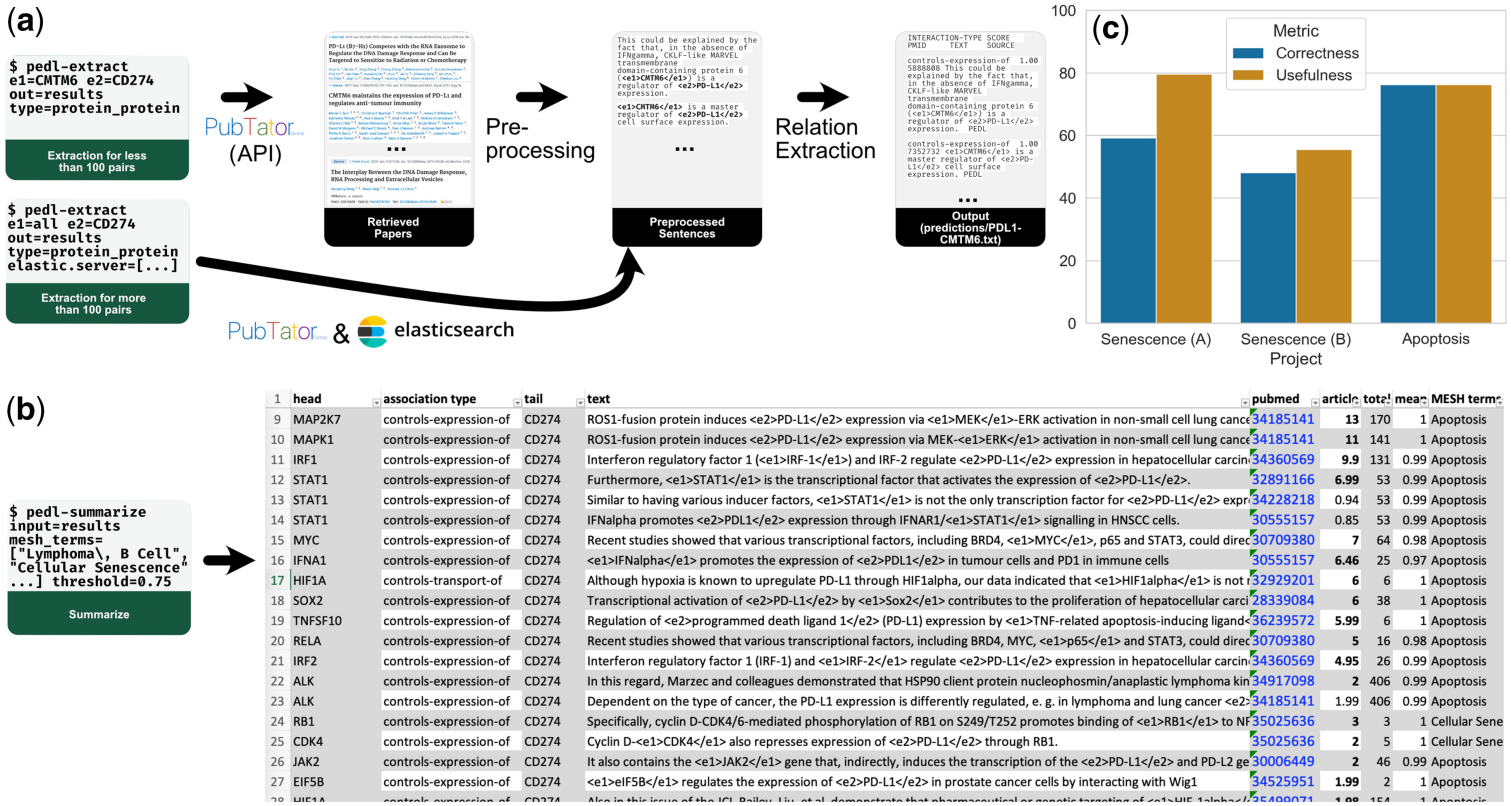
The main workflow using PEDL+’s two main commands extract (a) and summarize (b). (c) Results of our evaluation of PEDL+ in two pathway-curation projects. In (b) article score, total score and mean score are shortened to article, total, and mean.

We implement PEDL+ as a command-line application that allows easy installation and usage by non-experts. PEDL+ has two main commands: extract extracts PPAs/CPAs for a list of specified protein pairs or chemical–protein pairs which could result from experimental analysis or database searches. summarize allows to filter and interpret the results. Figure 1 shows a typical workflow.

The extract command extracts PPAs/CPAs (which collectively we call ‘relations’ from hereon) from a continuously updated version of the PubMed text mining subset (Comeau *et al.* 2019) for single or multiple entity pairs. For each entity pair (H,T), PEDL+ uses PubTator Central (PTC) (Wei *et al.* 2019) to retrieve all documents in which both entities of the pair occur together. For doing so, PEDL+ supports two modes: an API-based mode for small sets of entity pairs, and a local mode for larger sets. In the API-based mode, it uses PTC’s web API (https://www.ncbi.nlm.nih.gov/research/pubtator/api.html) to retrieve the text and the annotations of the respective articles. PEDL+ segments each of the texts into sentences with segtok (https://github.com/fnl/segtok) and selects those sentences where both entities occur. This mode is very useful for smaller entity sets as it requires no further installations, but is too slow for larger (n>100) sets. For the much faster local mode, PEDL+ maintains a preprocessed version of all PTC texts in an ElasticSearch (https://www.elastic.co/de/downloads/elasticsearch) index that can be (re-)built with the command rebuild_pubtator_index.

After retrieval and preprocessing, PEDL+ marks both entities with special marker tokens, as illustrated in Fig. 1a. If there are then multiple mentions per entity, PEDL+ will generate a copy of the sentence for every possible combination of mentions. Next, PEDL+ feeds each preprocessed sentence (see Fig. 1a for an example) through the relation model, which classifies the types of relation that the sentence expresses. For PPAs, we use an updated version of the RE model described in Weber *et al.* (2020). It is a version of the PLM SciBERT (Beltagy *et al.* 2019) fine-tuned on a mixture of gold-standard data and distantly supervised data derived from multiple pathway databases.

For CPAs, we apply a model derived from Weber *et al.* (2022). It is a version of the PLM LinkBERT-base (Yasunaga *et al.* 2022), fine-tuned on the DrugProt shared task dataset (Miranda *et al.* 2021). Its performance is comparable to our winning contribution to the shared task (Weber *et al.* 2021). Both models are described in more detail in Supplementary SM 2.

PEDL+ stores its results in a folder as one easy-to-parse file per pair in which each line represents one extracted relation together with the text of the sentence, the PubMed ID of the article, and the confidence of the extraction. For judging the relations of a single pair, it is usually enough to inspect the respective file. However, when extracting relations for multiple pairs, this task quickly becomes overwhelming. Therefore, the summarize command allows to post-process and interpret all relations found by an extract command (see Fig. 1b). It can be used to sort and filter the relations by confidence and/or by topic of the article a sentence stems from as defined by its MeSH terms (https://www.nlm.nih.gov/mesh/introduction.html). For instance, when one studies the biology of B-cell lymphoma, results from developmental biology could be distracting and should be excluded. This can be achieved by passing mesh_terms=“Lymphoma, B-Cell”, which would restrict results to those from articles labeled with this MeSH term.

The output of summarize can either be a csv file or an xlsx spreadsheet, which is created on a per-article base. That means that it reports every relation only once per article, even if it was extracted multiple times from it. It contains three different types of scores per extracted relation: (i) the *article score* is an indication of how much information an article contains about the relation and is defined as the sum of all confidence scores for extractions of the association in the article that are higher than the user-defined threshold; (ii) the *total score* measures how well the entire literature supports the relation and is calculated by summing all confidence scores that are higher than the threshold; and (iii) the *mean score* is calculated by dividing the total score by the number of text spans in which the relation was detected. Formally, let $S_{ij}$ be the confidence of the RE model that text span *i* supports PPA *j* and $A_{k}$ the set of text spans that come from article *k*. Then, the scores are defined as


(1) $$\begin{array}{c} a_{kj}=\sum_{i\in A_{k}}S_{ij},\;(\text{article}\;\;\text{score}) \end{array},$$

(2) $$\begin{array}{cc} t_{j}=\sum_{i}S_{ij}, & (\text{total}\;\;\text{score}), \end{array}$$

(3) $$\begin{array}{cc} m_{j}=\frac{t_{j}}{n} & (\text{mean}\;\;\text{score}), \end{array}$$

where *n* is the number of text spans in which PEDL+ detected the relation with a confidence higher than the user-defined threshold.

## 3 Evaluation by case studies

For an evaluation of the CPA model, we refer to Weber *et al.* (2022), where a comparable model achieved an F1 score of 79.7% on a large gold-standard dataset and a recall of 88.8% in a knowledge-base-population task. Here, we present results from a detailed manual evaluation of the PPA model based on two projects (see Supplementary SM 3 for details). The first project aimed to integrate the processes of cellular senescence into an already established model of cell fate-relevant signaling processes in B-cell lymphoma (Thobe *et al.* 2021). The second project focused on the development of a model for apoptosis regulation.

In both projects, PEDL+ was used to find relevant PPA among the constituents of the respective pathways, to prioritize interactions for which substantial evidence exists. PEDL+ searches were done with and without MeSH terms to discern between disease/cell-specific and ubiquitous interactions, exploiting the hierarchical nature of the MeSH ontology. For example, a model for B-cell lymphomas should be based on literature for B cells or lymphoma.

For the evaluation, coauthors K.H. and F.K. used PEDL+ for the senescence project and annotated the same set of PPAs, whereas L.L. evaluated PEDL+’s usefulness for the apoptosis project using a different set of PPAs. For both projects, curators first created pairs of gene sets H and T together with MeSH terms describing the scope of the project. Next, they used PEDL+ to search for all PPAs that connect a protein H∈H and a protein T∈T. Evaluation of the results was performed using the spreadsheet summary sorted by the article score. All top-ranking PPAs for each query were rated by assigning one out of the three possible scores 0 (not useful), 0.5 (maybe useful), and 1 (useful). Additionally, the annotators indicated whether PEDL+’s extraction was correct, i.e. whether the article confirms the existence of the PPA, regardless of its usefulness for the specific project.

In total, three annotators rated 156 unique PPA extractions for 43 protein pairs. Results can be found in Fig. 1c. Curators found PPA extractions to be correct in 48.2–76.2% of the cases. Interestingly, all three curators rated quite some incorrectly extracted PPAs as useful, leading to higher usefulness scores, with values between 55.6% and 79.6%. For many of these cases, the annotators commented that PEDL+ correctly detected that there is a PPA, but that it assigned the wrong type, which makes the precise prediction wrong in terms of our evaluation while it might still be interesting. This effect was especially pronounced for Senescence (Annotator A), who found only 59.3% of the PPAs to be correctly extracted, but 79.6% of the PPAs to be helpful.

We also measured inter-annotator agreement between the annotations of K.T. and F.K. by calculating Cohen’s κ for both correctness and usefulness. The agreement was markedly higher for correctness with κ=0.49 than for usefulness with κ=0.3, which suggests that the usefulness of PPA extractions varies strongly with the individual preference of the curator, even for the same curation project. This finding is additionally supported by the large difference of over 20 percentage points between the two usefulness ratings in the senescence project.

Finally, we asked the annotators to provide reasons for annotating a PPA as incorrect or as unhelpful. Detailed results can be found in Supplementary SM 4. In general, 50% of the incorrect extractions were truly wrong, while 40% were incorrect only because of the type or the direction of a PPA. Regarding helpfulness, the most frequently mentioned reason for not being helpful was that a PPA existed but not directly between the two entities; such indirect interactions are not helpful for pathway curation, although they would probably be considered helpful in a data enrichment project. Other sources of errors were incorrect MeSH annotation and insufficient evidence for the described PPA.

## 4 Conclusion

PEDL+ is a user-friendly biomedical RE tool that allows to extract PPAs and CPAs from the biomedical literature with a small set of commands. PEDL+ results are highly customizable and easily integrated in data analysis pipelines.

In our evaluation of PEDL+ in two pathway-curation projects, three annotators reported that 59–80% of its extractions were helpful for the project. A valuable contribution for future work would be to quantitatively compare PPA extraction tools in terms of usefulness and effectiveness. This would require an elaborate human–computer interaction study that controls for curator background, expertise, and learning effects.

## Supplementary Material

btad603_Supplementary_Data

## Data Availability

The data underlying this article are available at https://github.com/leonweber/pedl/tree/master/paper/eval_data.
