# Supplementary material for: PEDL+: protein-centered relation extraction from PubMed at your fingertip
Source: Bioinformatics. 2023 Nov 9;39(11):btad603. doi: 10.1093/bioinformatics/btad603 (PMC10660277; doi:10.1093/bioinformatics/btad603)
Supplement: btad603_Supplementary_Data [file btad603_supplementary_data.pdf]

Supplement:
PEDL+: Protein-centered relation extraction from PubMed at your fingertip

Leon Weber, Fabio Barth, Leonie Lorenz, Fabian Konrath,
Kirsten Huska, Jana Wolf, Ulf Leser

1 Comparison to other Tools

We qualitatively compare PEDL+ to the PPA-extraction tools INDRA (Gyori *et al.*, 2017) and PPAXE (Castillo-Lara and Abril, 2018), as well as to the text-mining subset of the protein-protein-interaction database STRING (Szklarczyk *et al.*, 2021) in Table SM1. We estimate the speed by taking 100 random protein pairs from the interaction database SignaLink (Csabai *et al.*, 2022) and measure the speed of each tool in extracting PPAs for all of them. INDRA comes with multiple extraction models and we use REACH (Valenzuela-Escárcega *et al.*, 2018) because it's endorsed by INDRA's authors[1] and we found its installation relatively straight-forward. As INDRA does not natively support extracting PPAs for protein pairs of interest, we implemented a workflow similar to PEDL+ using only INDRA functions in a small Python script. For PPAXE, we use the provided web interface[2]. PPAXE requires the user to provide the ids of PubMed articles (PMIDs) from which it should extract PPAs. Because PPAXE does not have a lookup capability for these PMIDs, we use INDRA to find all PubMed articles in which at least one of the 100 protein pairs occurs together.

2 Details on the RE Models

The PPA model that we use for PEDL+ differs in a few aspects from the model that we described in Weber *et al.* (2020). First, we updated the training data by adding more distantly supervised data and by removing one directly supervised dataset to improve the consistency of the annotations. Specifically, in addition to PID, the training data now includes PPAs derived from the PathwayCommons representations of Panther (Mi and Thomas, 2009)[3] and Net-

[1] `https://indra-db.readthedocs.io/en/latest/`

[2] `https://compgen.bio.ub.edu/PPaxe/`

[3] `https://www.pathwaycommons.org/archives/PC2/v12/PathwayCommons12.panther.hgnc.txt.gz`

	Speed (s / pair) (approx.)	Filter by MeSH	PMID Lookup	Evidence Spans	RE Model	Interface
STRING	< 0.1	✗	✗	✗	co-occurrence	Web / File
PPAXE	991.8	✗	✗	✓	random forest	Web / Python / CLI
INDRA (Reach)	398.1	✓	✓	✓	multiple (no DL / PLM)	Python
PEDL+ (local)	1.5	✓	✓	✓	PLM	CLI / Python

Table SM 1: Comparison of different text mining tools for PPA extraction. Speed is measured in seconds per protein pair estimated on a sample of 100 random related protein pairs. Filter by MeSH means whether the tool allows to filter results based on the MeSH terms of the articles in which the PPA was found. PMID lookup refers to the ability to find the PubMed articles in which two proteins occur together. Evidence span denotes the tools that provide the text snippet supporting the PPA for quick verification by a user. The following abbreviations are used: deep learning (‘DL’), PLM (‘pre-trained language model’), web interface (‘Web’), Python library (‘Python’), command-line interface (‘CLI’).

path (Kandasamy *et al.*, 2010)[4]. From all used databases we include the two additional PPA types `interacts-with`, which represents physical protein-protein interactions observed in high-throughput experiments, and `catalysis-precedes`, which is annotated when the head protein controls a reaction with a product that is used as substrate in another reaction controlled by the tail protein[5]. For the directly supervised data, we removed the BioNLP Epigenetics dataset, because its annotation guidelines are very different from those of the other datasets which led to the inclusion of many false negative annotations when combining all. Additionally, we made the MyGeneInfo-based normalization more lenient by introducing support for non-SwissProt proteins and for protein mentions that are mapped to more than one uniprot id, which allows mapping a larger fraction of gene mentions to uniprot ids. As we only include PPAs for proteins which can be resolved to uniprot, we obtain more PPAs per dataset, and thus, in the end, we have more directly supervised PPAs than in the dataset described in Weber *et al.* (2020). See Table 2, for statistics on the updated dataset.

We additionally introduced some minor modifications to the model architecture described in (Weber *et al.*, 2020). First, we use the concatenation of the final-layer embeddings of the entity start markers `<e1>` and `<e2>` instead of `[CLS]` to represent a text span for subsequent classification, because Baldini Soares *et al.* (2019) suggest that this can lead to more accurate extractions. Also, we use LogSumExp instead of maximum to aggregate the scores from the score matrix to form the evidence prediction to benefit from better gradient flow.

For CPAs, we retrain a model on the DrugProt shared task data (Miranda

[4] `https://www.pathwaycommons.org/archives/PC2/v12/PathwayCommons12.netpath.hgnc.txt.gz`

[5] `https://www.pathwaycommons.org/pc2/formats` (accessed 2022/09/22) and `https://www.biopax.org/owldoc/Level3/classes/MolecularInteraction___1004444555.html` (accessed 2022/09/22)

	Relations							
	expr.	phosph.	state	transport	complex	interacts	catalysis	total
Direct	412	196	334	107	918	0	0	1967
Distant	2646	6954	20732	1276	17718	2357	1353	53036

	Pairs		Spans (Avg.)	
	pos.	neg.	pos.	neg.
Direct	1563	4689	17.7	5
Distant	42768	531557	36.7	3.8

Table SM 2: 'Pairs' is the total number of protein pairs with at least one PPA (pos.) and without any PPA (neg.). 'Spans' states the average number of text spans per protein pair for pairs with at least one PPA (pos.) and without any PPA (neg.). Abbreviations: 'expr.' - controls-expression-of, 'phosph.' - controls-phosphorylation-of, 'state' - controls-state-change-of, 'transport' - controls-transport-of, 'interacts' - interacts-with, 'catalysis' - catalysis-precedes

et al., 2021) that is based on the single-model baseline without entity descriptions in Weber *et al.* (2022). Unfortunately, the licensing of *RoBERTa-large-PM-M3-Voc* (the strongest base model in our evaluation in Weber *et al.* (2022)) prohibits commercial use. Thus we replace it with LinkBert-base (Yasunaga *et al.*, 2022) because of its strong performance in BioNLP tasks. We fine-tune it for 3 epochs on the training portion of DrugProt and obtain an F1 score of 78.7% on the development set, which is comparable with the best single-model configuration reported in Weber *et al.* (2022).

3 Evaluation Projects

In the first project, two curators sought to develop models based on ordinary differential equations and Boolean logic that describe the role of cellular senescence in B-cell lymphoma. We provide the gene sets and results for both projects as supplementary files. For this, they used PEDL+ to connect a recently proposed transcriptomic signature for cellular senescence in diffuse large B-cell lymphoma patients (Reimann *et al.*, 2021) to inhouse models of B-cell development based on the models of Roy *et al.* (2019) and Thobe *et al.* (2021). Here, we used the MeSH filter `Lymphoma, B-Cell`.

In the second project, a third curator developed a Boolean model for the intrinsic pathway of apoptotic regulation with a specific focus on the role of the BCL-2 family. They used PEDL+ to extract PPAs in two ways; (1) among 15 different members of the BCL-2 family and (2) between these BCL-2 family members and a list of putative upstream regulators of apoptosis based on the models of Roy *et al.* (2019) and thobePatientSpecificModelingDiffuse2021. In this project, we provided the annotator with results without any MeSH filter

and, additionally, results for (1) filtered by the MeSH term `Lymphoma, B-Cell`.

4 Error Analysis

The results of the error analysis can be found in Figure SM 1. The most frequently cited reasons for incorrect PPA extractions were that PEDL erroneously extracted a PPA from a sentence that does not state it and that it assigned the wrong type for a PPA. For the unhelpful PPAs, the results are inconclusive because annotators provided only 16 annotations in total and the numbers are close together. Cited reasons were that articles discussed the PPA in the context of a disease that is irrelevant to the curation context, that the extracted PPA is known to be an indirect interaction, that the article did not provide sufficient biochemical evidence for the PPA, and that the PPA is only true in specific contexts, e.g. when a protein is mutated or a drug is administered.

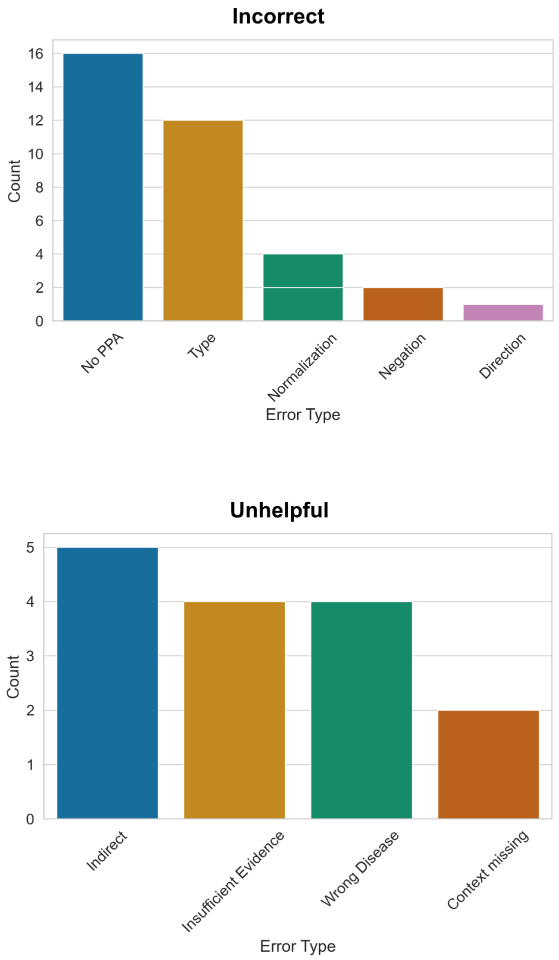

Figure SM 1: Results of the error analysis for incorrect (top) and unhelpful (bottom) PPAs. **Incorrect:** 'No PPA' means that the article does not confirm the PPA, 'Type' that the article states a PPA for the correct protein pair, but the wrong type of PPA was extracted. 'Normalization' refers to cases in which the PPA was correctly extracted but PubTator Central assigned at least one wrong gene id for the pair. 'Negation' describes cases in which the two proteins and the PPA type are correct, but the existence of the PPA is explicitly negated in the article. 'Direction' means that the head and tail of the PPA should be inversed. **Unhelpful:** 'Indirect' means that the interaction is indirect but the curator was interested only in direct interactions, 'insufficient evidence' means that there was not enough biochemical evidence to support the plausibility of the PPA, 'wrong disease' refers to cases in which the PPA was specific to a disease that is irrelevant to the curation context and 'context missing' to cases where the PPA is only valid in certain contexts such as when the protein is mutated or a drug is administered.

References

Baldini Soares, L., FitzGerald, N., Ling, J., and Kwiatkowski, T. (2019). Matching the Blanks: Distributional Similarity for Relation Learning. In *Proceedings of the 57th Annual Meeting of the Association for Computational Linguistics*, pages 2895–2905, Florence, Italy. Association for Computational Linguistics.

Castillo-Lara, S. and Abril, J. F. (2018). PPaxe: Easy extraction of protein occurrence and interactions from the scientific literature. *Bioinformatics (Oxford, England)*, **35**(14), 2523–2524.

Csabai, L., Fazekas, D., Kadlecsik, T., Szalay-Bekő, M., Bohár, B., Madgwick, M., Módos, D., Ölbei, M., Gul, L., Sudhakar, P., Kubisch, J., Oyeyemi, O. J., Liska, O., Ari, E., Hotzi, B., Billes, V. A., Molnár, E., Földvári-Nagy, L., Csályi, K., Demeter, A., Pápai, N., Koltai, M., Varga, M., Lenti, K., Farkas, I. J., Türei, D., Csermely, P., Vellai, T., and Korcsmáros, T. (2022). SignaLink3: A multi-layered resource to uncover tissue-specific signaling networks. *Nucleic Acids Research*, **50**(D1), D701–D709.

Gyori, B. M., Bachman, J. A., Subramanian, K., Muhlich, J. L., Galescu, L., and Sorger, P. K. (2017). From word models to executable models of signaling networks using automated assembly. *Molecular Systems Biology*, **13**(11), 954.

Kandasamy, K., Mohan, S. S., Raju, R., Keerthikumar, S., Kumar, G. S. S., Venugopal, A. K., Telikicherla, D., Navarro, J. D., Mathivanan, S., Pecquet, C., Gollapudi, S. K., Tattikota, S. G., Mohan, S., Padhukasahasram, H., Subbannayya, Y., Goel, R., Jacob, H. K. C., Zhong, J., Sekhar, R., Nanjappa, V., Balakrishnan, L., Subbaiah, R., Ramachandra, Y. L., Rahiman, B. A., Prasad, T. S. K., Lin, J.-X., Houtman, J. C. D., Desiderio, S., Renauld, J.-C., Constantinescu, S. N., Ohara, O., Hirano, T., Kubo, M., Singh, S., Khatri, P., Draghici, S., Bader, G. D., Sander, C., Leonard, W. J., and Pandey, A. (2010). NetPath: A public resource of curated signal transduction pathways. *Genome Biology*, **11**(1), R3.

Mi, H. and Thomas, P. (2009). PANTHER Pathway: An Ontology-Based Pathway Database Coupled with Data Analysis Tools. In Y. Nikolsky and J. Bryant, editors, *Protein Networks and Pathway Analysis*, Methods in Molecular Biology, pages 123–140. Humana Press, Totowa, NJ.

Miranda, A., Mehryary, F., Luoma, J., Pyysalo, S., Valencia, A., and Krallinger, M. (2021). Overview of DrugProt BioCreative VII track: Quality evaluation and large scale text mining of drug- gene/protein relations. *Proceedings of the BioCreative VII challenge evaluation workshop*, page 11.

Reimann, M., Schrezenmeier, J., Richter-Pechanska, P., Dolnik, A., Hick, T. P., Schleich, K., Cai, X., Fan, D. N. Y., Lohneis, P., Maßwig, S., Denker, S., Busse, A., Knittel, G., Flümann, R., Childs, D., Childs, L., Gätjens-Sanchez, A.-M., Bullinger, L., Rosenwald, A., Reinhardt, H. C., and Schmitt,

C. A. (2021). Adaptive T-cell immunity controls senescence-prone MyD88- or CARD11-mutant B-cell lymphomas. *Blood*, **137**(20), 2785–2799.

Roy, K., Mitchell, S., Liu, Y., Ohta, S., Lin, Y.-S., Metzig, M. O., Nutt, S. L., and Hoffmann, A. (2019). A Regulatory Circuit Controlling the Dynamics of NFκB cRel Transitions B Cells from Proliferation to Plasma Cell Differentiation. *Immunity*, **50**(3), 616–628.e6.

Szklarczyk, D., Gable, A. L., Nastou, K. C., Lyon, D., Kirsch, R., Pyysalo, S., Doncheva, N. T., Legeay, M., Fang, T., and Bork, P. (2021). The STRING database in 2021: Customizable protein–protein networks, and functional characterization of user-uploaded gene/measurement sets. *Nucleic acids research*, **49**(D1), D605–D612.

Thobe, K., Konrath, F., Chapuy, B., and Wolf, J. (2021). Patient-Specific Modeling of Diffuse Large B-Cell Lymphoma. *Biomedicines*, **9**(11), 1655.

Valenzuela-Escárcega, M. A., Babur, Ö., Hahn-Powell, G., Bell, D., Hicks, T., Noriega-Atala, E., Wang, X., Surdeanu, M., Demir, E., and Morrison, C. T. (2018). Large-scale automated machine reading discovers new cancer-driving mechanisms. *Database: The Journal of Biological Databases and Curation*, **2018**.

Weber, L., Thobe, K., Migueles Lozano, O. A., Wolf, J., and Leser, U. (2020). PEDL: Extracting protein–protein associations using deep language models and distant supervision. *Bioinformatics*, **36**(Supplement_1), i490–i498.

Weber, L., Sänger, M., Garda, S., Barth, F., Alt, C., and Leser, U. (2022). Chemical-Protein Relation Extraction with Ensembles of Carefully Tuned Pretrained Language Models. *Database*, page (accepted).

Yasunaga, M., Leskovec, J., and Liang, P. (2022). LinkBERT: Pretraining language models with document links. In *Proceedings of the 60th Annual Meeting of the Association for Computational Linguistics (Volume 1: Long Papers)*, pages 8003–8016, Dublin, Ireland. Association for Computational Linguistics.
